# Therapeutic outcomes of endoscopic papillectomy for ampullary neoplasms: retrospective analysis of a multicenter study

**DOI:** 10.1186/s12876-017-0626-5

**Published:** 2017-05-30

**Authors:** Sung Hoon Kang, Kook Hyun Kim, Tae Nyeun Kim, Min Kyu Jung, Chang Min Cho, Kwang Bum Cho, Ji Min Han, Ho Gak Kim, Hyun Soo Kim

**Affiliations:** 1 Division of Gastroenterology and hepatology, Department of Internal Medicine, Yeungnam University College of Medicine, 317-1 Daemyung-dong, 705-717 Nam-gu Daegu, South Korea; 2 Department of Internal Medicine, Kyungpook National University School of Medicine, Daegu, South Korea; 3 Department of Internal Medicine, Keimyung University School of Medicine, Daegu, South Korea; 4 Department of Internal Medicine, Catholic University of Daegu School of Medicine, Daegu, South Korea; 5 Department of Internal Medicine, Daegu Fatima Hospital, Daegu, South Korea

**Keywords:** Ampullary neoplasms, Endoscopic papillectomy

## Abstract

**Background:**

Endoscopic papillectomy (EP) is reported to be a relatively safe and reliable procedure for complete resection of ampullary neoplasms. The aim of this study was to evaluate the therapeutic outcomes and complications of EP for ampullary neoplasms.

**Methods:**

A retrospective multicenter study was conducted with 5 participating centers from January 2007 to July 2014. A total of 104 patients who underwent EP for ampullary neoplasms were reviewed retrospectively. EP was performed by snare resection with or without submucosal lifting of the lesion.

**Results:**

The mean age of patients was 60.5 ± 12.1 years, and the male-to-female ratio was 2.0:1. En bloc resection was possible in 94 patients (90.3%). A biliary and a pancreatic stent were placed after EP in 42 patients and in 60 patients, respectively. A pathologically incomplete resection was noted in 11 cases (10.6%), and 5 of these patients were treated with additional endoscopic procedure. Histology of resected specimens was as follows: low grade adenoma (43.2%), high grade adenoma (14.4%), adenocarcinoma (16.3%), hyperplastic polyp (7.7%), and others (18.4%). Of the 75 cases with low grade adenoma on biopsy specimen, 21.3% turned out to have high grade adenoma (12%) or adenocarcinoma (9.3%). Procedure-related complications occurred in 33 patients (31.7%); bleeding (18 cases, 17.3%), pancreatitis (16 cases, 15.4%), and perforation (8 cases, 7.7%). Pre-EP ERCP, saline lifting, sphincterotomy, biliary stenting, pancreatic stenting, specimen size, and cauterization were not related to post EP complications. Surgery was performed in 6 cases with pathological incomplete resection and 2 cases with complications after EP, and there were 2 cases of mortality due to complications. During follow-up endoscopy after initial success of EP, remnant tumors were found in 7 patients, one of whom underwent surgery and the others were treated endoscopically. Consequently, the overall endoscopic success rate of EP was 89.4%.

**Conclusions:**

Endoscopic papillectomy appears to be an effective treatment for ampullary neoplasms, and can be considered as an alternative to surgery. However, relatively high risk of procedure related complications is a problem that must be considered.

## Background

Ampullary neoplasms, usually found in the sixth to eighth decades of life [1, 2], represent less than 5% of all new digestive tract neoplasms [3]. However, they are being identified more frequently with the increasing application of esophagogastroduodenoscopy (EGD) and endoscopic retrograde cholangiopancreatography (ERCP).

Patients with ampullary neoplasm presented with biliary colic, obstructive jaundice, and nonspecific symptoms including weight loss, vague abdominal pain, dyspepsia, malaise, and anorexia; however, most ampullary neoplasms are found incidentally during EGD. Complete resection is required for ampullary neoplasms, whether malignant or not, due to the potential for malignant transformation. Like their histological and genetic similarities to colon adenomas, ampullary neoplasms are known to follow an adenoma-to-carcinoma sequence [4–6]. Due to the high rate of discrepancy for pathologic results before and after endoscopic resection, ranging from 25% to 60% [7, 8], complete excision is mandatory for accurate diagnosis as well as treatment.

Recently, endoscopic papillectomy has been reported to be a relatively safe and reliable method for complete resection of ampullary neoplasms [9–12]. However, few large-scale studies concerning the effectiveness and complications related to EP have been reported. The aim of this study was to evaluate the therapeutic outcomes and complications of EP for ampullary neoplasms.

## Methods

A total of 104 patients in 5 participating centers who underwent endoscopic papillectomy for treatment of ampullary neoplasm from January 2007 to July 2014 were reviewed retrospectively. All papillary tumors were benign on initial forcep biopsy or confined to mucosa on endoscopic ultrasonogram (EUS) in cases of adenocarcinoma. When carcinoma was demonstrated on forcep biopsy or suspected on endoscopic findings, abdominal computerized tomogram (CT) scan, endoscopic retrograde cholangiopancreatography (ERCP), and EUS were performed to evaluate for advanced or metastatic disease. ERCP was obtained to confirm the intraductal growth of the tumor and EUS was performed to determine depth of tumor within the duodenal wall before snare papillectomy in some patients. Patients with a suspicious direct biliary or pancreatic extension of the lesion, invasive carcinoma, metastatic disease, or coagulopathy did not undergo EP. Patients who were lost to follow-up for at least 6 months after EP were excluded. The baseline characteristics of the patients, the technique of endoscopic papillectomy, the pathologic findings, and the clinical outcomes were evaluated. This study was approved by the institutional review board of each institution. Written informed consent was obtained from all patients prior to the procedure.

The ampulla should first be inspected for macroscopic signs of advanced malignancy: firmness, ulceration, induration, and friability. EP was performed by snare resection with or without submucosal lifting of the lesion after visualization of the macroscopic appearance of the tumors. A standard polypectomy snare was used and blended electrical current (50–60 J) was applied. After snaring the tumor downward or upward, constant tension was applied to the snare loop until the lesion was completely resected. For lesions that did not permit an en bloc resection, piecemeal resection was performed for complete removal of the tumor. Argon plasma coagulation (APC) was done for fulguration of small tumor remnants unfit for repeated snare resection. Selection of treatment modality for suspicious remnant tumors was determined by availability and experience of endoscopists. The resection site for any residual tumor and complications were thoroughly evaluated at the end of procedures. A pancreatic stent and a biliary stent were placed immediately after excision of the tumor at the discretion of the endoscopists in some patients. Endoscopic sphincterotomy (EST) of the bile duct was performed when clinically indicated. A pancreatogram and a cholangiogram were obtained to ensure adequate pancreatobiliary drainage. The resected specimens were extracted using a basket or forcep.

Outcome parameters were categorized as endoscopic success, endoscopic failure, and complications. Endoscopic success was defined as a complete excision of the lesion, absence of pathological incomplete resection, and absence of remnant tumor during follow up. Pathological incomplete resection, recurrence and remnant cases that were treated endoscopically were regarded as endoscopic success. Cases with pathological incomplete resection, recurrence or remnant cases or complications requiring surgery or mortality cases were defined as endoscopic failure. Tumor cell positivity at follow-up endoscopy within 6 months is defined as remnant tumor and positive-tumor cells at follow-up endoscopy over 6 months after EP is defined as tumor recurrence [13, 14].

Complications of papillectomy were arbitrarily defined as early and late: (1) pancreatitis, bleeding and perforation belong to early complication (2) post-EP stenosis belongs to late complication. For cases with pathologically complete resection, surveillance endoscopies were performed at regular intervals. Endoscopic biopsy was performed when polypoid or ulcerative lesions were observed at follow-up. Additional management such as EP, APC or surgery was performed when the biopsy demonstrated any adenomatous tissue. Surgery was recommended for all cases with remnant cancer cells after endoscopic therapy. The indications for surgery were as follows: histopathologic documentation of persistent high-grade dysplasia, co-existent carcinoma, and intraductal extension of the adenoma. However, patients who refused or were unfit for surgery underwent additional snare excision or APC.

### Statistical analysis

Statistical analyses were performed using PASW 18.0 statistical software (PASW, USA). Risk factors of complication were performed using the chi-square test and the independent Student’s *t* test. Multivariate analysis for the risk factors of complication was performed using logistic regression. A p-value less than 0.05 was considered statistically significant.

## Results

The mean age of the 104 patients was 60.5 ± 12.1 years (range 37–86), and the male-to-female ratio was 2.0:1. Of the total 104 patients, 63 patients (60.6%) had associated diseases; diabetes mellitus, ischemic heart disease, liver cirrhosis, and chronic kidney disease, and others. Ninety three patients (89.4%) were classified as 1 or 2 according to the American Society of Anesthesiology (ASA) classification, and 79 patients (75.9%) were asymptomatic at diagnosis. The mean duration of hospital stay was 5.4 days (range 1–30) and the mean follow-up period after EP was 44.2 months (range 6–90) (Table 1).

**Table 1:** Basic characteristics of patients

	N (%) (*n* = 104)
Age (yrs)	60.5 ± 12.1 (37–86)
M:F (ratio)	69:35 (2.0:1)
BMI (kg/m^2^)	24.2 ± 3.0
Associated diseases	
Diabetes mellitus	17 (16.3)
Ischemic heart disease	7 (6.7)
Liver cirrhosis	2 (1.9)
Chronic kidney disease	1 (0.9)
ASA score (1/2/3/4)	46/47/10/1 (44.2/45.2/9.6/0.9)
Symptoms	
No symptom	79 (75.9)
Abdominal discomfort	17 (16.3)
Jaundice	1 (0.9)
Mean hospital stay (days)	5.4 (1–30)
Mean follow-up (months)	44.2 (6–90)

*ASA* American society of anesthesiology. Values are presented as mean ± SD, number (%), or number (range)

ERCP (79.8%), CT (53.8%), EUS (38.5%), magnetic resonance cholangiopancreatography (MRCP) (3.9%), or positron emission tomography (PET) CT (0.9%) was used in further evaluations of ampullary lesions. Pre-EP cholangiogram and pancreatogram were obtained in 79 (75.9%) and 73 patients (70.1%), respectively. Saline or a dilute solution of epinephrine (1:10,000) was injected submucosally to lift the lesion before EP in 37 patients (35.6%). En bloc resection was possible in 94 (90.3%) and piecemeal resection in 10 patients (9.7%). Adjunctive cauterization with APC or multipolar electrocoagulation was applied to ablate any possible remnant tumor at the margin of resection after EP in 36 patients (34.6%). A biliary and a pancreatic sphincterotomy were performed in 56 (53.8%) and 24 (23.1%) patients, respectively. After EP, 42 patients (40.1%) had biliary stenting and 60 (57.7%) had pancreatic stenting (Table 2).

**Table 2:** Techniques of endoscopic papillectomy

	N (%) (*n* = 104)
Pre papillectomy ERCP	
Cholangiogram	79 (75.9)
Pancreatogram	73 (70.1)
Submucosal lifting	37 (35.6)
Types of resection	
En bloc	94 (90.3)
Piecemeal	10 (9.7)
Cauterization after resection	36 (34.6)
Sphincterotomy	
Bile duct	56 (53.8)
Pancreatic duct	24 (23.1)
Stenting	
Biliary	42 (40.1)
Pancreatic	60 (57.7)

*ERCP* endoscopic retrograde cholangiopancreatography. Values are presented as numbers (%)

Mean size of the resected specimens was 13.5 ± 5.9 mm (range 2–32). Histology of resected specimen was as follows: low grade adenoma (43.2%), high grade adenoma (14.4%), adenocarcinoma (16.3%), hyperplastic polyp (7.7%), and others (18.4%). The concordance rate of pathologic diagnosis between the endoscopic forceps biopsy and the resected specimen was 60.6% (57/94) according to the Vienna classification. Underestimated diagnosis was 20.2% (19/94) and overestimated diagnosis was 19.2% (18/94). Of the 75 cases with low grade adenoma on forcep biopsy, 16 cases (21.3%) turned out to have high grade adenoma (9 cases, 12%) or adenocarcinoma (7 cases, 9.3%) (Table 3).

**Table 3:**
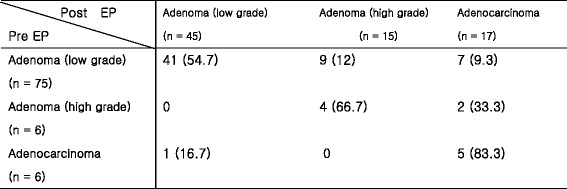
Comparison of pathologic findings of pre- and post-endoscopic papillectomy

*EP* Endoscopic papillectomy. Values are presented as numbers (%)

A pathologically incomplete resection was noted in 11 cases (10.6%) (Table 4). Of the 11 cases of pathologically incomplete resection, five patients were treated with additional endoscopic procedures and the other 6 cases were referred to surgery for complete resection. Procedure-related complications occurred in 33 patients (31.7%): bleeding (18 cases), pancreatitis (16 cases), and perforation (8 cases). Of the 18 cases of bleeding, 17 patients improved with medical treatment and 1 patient underwent surgical management. All patients with pancreatitis recovered conservatively. Of the 8 cases of perforation, 5 patients improved with conservative management, however 3 patients required surgery. Procedure-related death occurred in two patients. One patient was classified as 3 or 4 according to the American Society of Anesthesiology (ASA) classification and pre-EP diagnosis was adenocarcinoma. After EP, the patient underwent an open laparotomy due to retroperitoneal perforation complicated by ongoing bleeding, which contributed directly to his death. Another patient was classified as 1 or 2 according to ASA classification and pre-EP diagnosis was low grade adenoma. After EP, the patient underwent percutaneous drainage and medical treatment due to retroperitoneal perforation. However, he died as the result of an aggravation of infection (Table 5). Pre-EP ERCP, saline lifting, sphincterotomy, biliary stenting, pancreatic stenting, specimen size, and cauterization were not related to post EP complications (Table 6). The incidence of pancreatitis was not significantly different regardless of the pancreatic stenting (Table 7). Remnant tumors were detected in 7 patients on follow-up endoscopy, six of whom were treated endoscopically and one patient was referred to surgery (Table 8).

**Table 4:** Pathologic findings of resected specimens

	N (%)
Resection specimen size (mm)	13.5 ± 5.9 (2–32)
Pathologic findings	
Adenocarcinoma	17 (16.3)
Adenoma	
Low grade	45 (43.2)
High grade	15 (14.4)
Hyperplastic polyp	8 (7.7)
Inflammation	8 (7.7)
Adenomyoma	4 (3.8)
Carcinoid	3 (2.9)
Lymphoma	1 (0.9)
Resection margin	
Negative	93 (89.4)
Positive	11 (10.6)

Values are presented as mean ± SD, numbers (%) or number (range)

**Table 5:** Complications of endoscopic papillectomy and outcomes of management

	N (%)	Management (Medical/Operation)	Recovery (%)
Bleeding	18 (17.3)	17/1	18 (100)
Pancreatitis	16 (15.4)	16/0	16 (100)
Perforation	8 (7.7)	6/2	6 (75)
Mortality	2 (1.9)^a^		

^a^One case was caused by retroperitoneal perforation complicated with persistent bleeding. Another was also caused by retroperitoneal perforation combined with ongoing infection

**Table 6:** Statistical analysis of risk factors of complication

	Pancreatitis (p-value)	Bleeding (p-value)	Perforation (p-value)
Age	0.404	0.314	0.656
Sex	0.139	0.606	0.317
BMI	0.077	0.137	0.343
Pancreatogram	0.117	0.073	0.289
P-duct stenting	0.137	0.177	0.648
B-duct stenting	0.397	0.363	0.198
APC	0.383	0.676	0.858
Sphincterotomy	0.409	0.315	0.777
Saline lifting	0.196	0.165	0.382
Tumor size	0.935	0.434	0.670

*BMI* body mass index. *APC* Argon plasma coagulation

**Table 7:** Frequency of pancreatitis according to the presence of pancreatic stent

	With pancreatic stent (*n* = 60)	Without pancreatic stent (*n* = 44)
Pancreatitis (+)	12 (20%)	4 (9.1%)
Pancreatitis (−)	48 (80%)	40 (90.9%)

Values are presented as numbers (%)

**Table 8:** Management of patients with pathologically incomplete resection and remnant tumors at follow-up

	Pathologically incomplete resection (*n* = 11)	Remnant tumors at follow-up (*n* = 7)
Endoscopic papillectomy	0	3
APC	4	2
Endoscopic papillectomy with APC	1	1
Operation	6	1

*APC* Argon plasma coagulation

Eleven patients had endoscopic failure; 9 patients underwent surgery due to pathological incomplete resection (6 cases), remnant tumor during follow up (1 case), and complications (2 cases) and 2 patients died due to complications. Of the remaining 93 cases of ampullary neoplasm treated endoscopically, no recurrences were found during a mean follow-up period of 44.2 months. Consequently, the overall endoscopic success rate of EP was 89.4% (93/104).

## Discussion

Pancreaticoduodenectomy or local surgical resection has been considered as a treatment of choice for ampullary neoplasms. However, perioperative mortality occurs in 4-15% and morbidity in up to 50% following surgical resection [15–17]. Even local resection has a reported morbidity rate of 19- 25% with recurrence rate up to 32% at 5 years after surgery [18]. Endoscopic papillectomy (EP) was first reported by Binmoeller et al. [19] in 1993 with the promise of lower morbidity and mortality. Since then, endoscopic papillectomy with curative intent has emerged as an effective alternative to Whipple’s procedure for management of uncomplicated ampullary neoplasms [19–23].

In a study on a combined 4-center experience in 103 patients with ampullary neoplasms undergoing endoscopic papillectomy, success rate up to 80% of patients, with a 20% recurrence rate and a 10% complication rate were reported (Catalano et al. [13]). Another study reported a success rate of EP of 73%, the complication rate was 15%, and recurrence rate was 15% (Bohnacker et al. [24]). In the current study, the overall success rate of EP was 89.4%, the complication rate was 31.7%, and there was no recurrence after 6 months following successful EP. Differences in the success rate and complications of endoscopic papillectomy in studies may be related to differences in the inclusion criteria for EP and various procedural factors in performing EP among studies.

Close and serial follow-up after endoscopic papillectomy are required for identification of remnant tumors or recurrence. Previous studies [13] [14] reported that all recurrences were found within the first 14 months of papillectomy, with distinguishing a recurrent ampullary lesion from remnant tissue with evidence of a positive biopsy result. Catalano et al. [13] proposed guidelines for performing endoscopic surveillance every 6–12 months after successful EP for at least 2 years. In the current study, 7 cases of remnant tumors were found within 6 months of follow up after initial EP and only 1 case required surgery, and there were no recurrences during long term follow-up after 6 months in 93 cases with endoscopic success. Based on our result, if no clinical evidence of recurrence was found within 6 months of follow up, possibility of recurrence was very low and further follow-up can be individualized.

Careful selection of patients with proper indications of EP for ampullary neoplasm is important for the success of EP. Although 4 out of 6 cases with adenocarcinomas were treated successfully with endoscopic papillectomy without recurrence, EP was not generally recommended in cases of adenocarninoma due to potential risk of regional lymph node metastasis even in case of earlier stage. The false-negative rates for endoscopic biopsies are reported to range from 25 to 60%, indicating that an accurate differentiation of malignant from benign lesions cannot be based solely on a pathologic report of biopsy specimens [7, 8]. In the current study, the concordance rate of biopsy specimens with post EP specimens was only 60.6% (57/94). Careful examination and multiple biopsies of the ampullary lesions at initial endoscopy are strongly recommended to increase the histologic yield and reduce false negative rates.

Despite the overall success rate of EP of almost 90%, complications associated with EP are a problem that must be considered and careful observation is required after EP for any possible complications. Significant procedure-related complications including hemorrhage, pancreatitis, and perforation occurred in 31.7% of patients, and of these there were 2 cases of mortality. Development of papillary stenosis was reported as a late complication of EP, but there were no cases of clinically significant papillary stenosis in this study. Pre-EP ERCP, saline lifting of the lesion before EP, pancreatic or biliary sphincterotomy, biliary or pancreatic stenting, size of the specimen, and cauterization were not related to post EP complications.

Post EP perforation was the most serious complication, and 2 mortality cases were related to perforation. Although risk of perforation is likely to increase with the tumor size, there was no way to predict perforation in patients undergoing EP. Early recognition of perforation and proper treatment with either endoscopic or surgical management are mandatory to avoid subsequent deterioration of the patients due to perforation.

Development of pancreatitis is a common and problematic complication after EP. Currently, several studies have shown that placement of a prophylactic pancreatic stent after EP reduces the risk of pancreatitis [23, 25, 26]. A prospective, randomized, controlled trial showed that pancreatic duct stenting after EP significantly decreased the rate of post-procedure [26]. However, whether or not post EP pancreatic stenting can alleviate the rate of pancreatitis is still controversial. Some studies have shown no statistically significant benefit by prophylactic placement of a pancreatic stent during EP [6, 13]. In the current study, pancreatic stenting showed no statistically significant benefit by prophylactic placement of a pancreatic stent during EP. An increase in the difficulty of the pancreatic duct cannulation after EP and the need for greater manipulation of the papilla may increase the risk of pancreatitis in patients with prophylactic placement of a pancreatic stent. Further prospective controlled studies are required to evaluate the efficacy of prophylactic pancreatic stenting after EP for prevention of post EP pancreatitis.

There were several limitations in this study. Defining the exact inclusion criteria for EP was difficult and pre EP study differed in each institution and each case because of retrospective design of this study. Appropriate indications of EP for ampullary neoplasm and pre EP evaluation are important. Evaluation of risk factors of complications associated with EP is difficult due to small sample size. Evaluation of risk factors associated with post EP complications is very important because considerable risk was reported after EP as in the current study. More studies concerning complications related to EP are needed.

## Conclusions

In conclusion, EP appears to be an effective treatment for ampullary neoplasm. However, relatively high risk of procedure related complications is a problem that must be considered. Considering the morbidity and mortality of surgical resection, endoscopic approach can be an effective alternative option for management of uncomplicated ampullary neoplasms and in patients who are unfit for surgery due to poorer general condition. Careful selection of patients with appropriate criteria for EP or surgery is required for management of patients with ampullary neoplasm.

## Abbreviations

APC: Argon plasma coagulation
ASA: American Society of Anesthesiology
CT: Computerized tomogram
EGD: Esophagogastroduodenoscopy
EP: Endoscopic papillectomy
ERCP: Endoscopic retrograde cholangiopancreatography
EST: Endoscopic sphincterotomy
EUS: Endoscopic ultrasonogram
MRCP: Magnetic resonance cholangiopancreatography
PET: Positron emission tomography

## References

[1] Ryan DP, Schapiro RH, Warshaw AL (1986). Villous tumors of the duodenum. Ann Surg.

[2] Gray G, Browder W (1989). Villous tumors of the ampulla of Vater: local resection ersus pancreatoduodenectomy. South Med J.

[3] Scarpa A, Capelli P, Zamboni G (1993). Neoplasia of the ampulla of Vater. Ki-ras and p53 mutations. Am J Pathol.

[4] Stolte M, Pscherer C (1996). Adenoma-carcinoma sequence in the papilla of Vater. Scand J Gastroenterol.

[5] Chung CH, Wilentz RE, Polak MM (1996). Clinical significance of K-ras oncogene activation in ampullary neoplasms. J Clin Pathol.

[6] Cheng CL, Sherman S, Fogel EL, McHenry L, Watkins JL, Fukushima T, Howard TJ, Lazzell-Pannell L, Lehman GA (2004). Endoscopic snare papillectomy for tumors of the duodenal papillae. Gastrointest Endosc.

[7] Yamaguchi K, Enjoji M, Kitamura K (1990). Endoscopic biopsy has limited accuracy in diagnosis of ampullary tumors. Gastrointest Endosc.

[8] Seifert E, Schulte F, Stolte M (1992). Adenoma and carcinoma of the duodenum and papilla of Vater: a clinicopathologic study. Am J Gastroenterol.

[9] Cotton PB, Lehman G, Vennes J (1991). Endoscopic sphincterotomy complications and their management: an attempt at consensus. Gastrointest Endosc.

[10] Grace PA, Pitt HA, Tompkins RK (1986). Decreased morbidity and mortality after pancreaticoduodenectomy. Am J Surg.

[11] Cameron JL, Pitt HA, Yeo CJ (1993). One hundred and forty-five consecutive pancreaticoduodenectomies without mortality. Ann Surg.

[12] Matsumoto T, Iida M, Nakamura S (2000). Natural history of ampullary adenoma in familial adenomatous polyposis: reconfirmation of benign nature during extended surveillance. Am J Gastroenterol.

[13] Catalano MF, Linder JD, Chak A (2004). Endoscopic management of adenoma of the major duodenal papilla. Gastrointest Endosc.

[14] Irani S, Arai A, Ayub K (2009). Papillectomy for ampullary neoplasm: results of a single referral center over a 10-year period. Gastrointest Endosc.

[15] Wong RF, DiSario JA (2004). Approaches to endoscopic ampullectomy. Curr Opin Gastroenterol.

[16] Birkmeyer JD, Stukel TA, Siewers GPP, Wennberg DE, Lucas FL (2003). Surgeonvolume and operative mortality in the United States. N Engl J Med.

[17] Sarmiento JM, Thompson GB, Nagorney DM, Donohue JH, Farnell MB (2002). Pancreassparing duodenectomy for duodenal polyposis. Arch Surg.

[18] Farnell MB, Sakorafas GH, Sarr MG, Rowland CM, Tsiotos GG, Farley DR (2000). Villous tumors of the duodenum: reappraisal of local vs. extended resection. J Gastrointest Surg.

[19] Binmoeller KF, Boaventura S, Ramsperger K (1993). Endoscopic snare excision of benign adenomas of the papilla of Vater. Gastrointest Endosc.

[20] Park SW, Song SY, Chung JB (2000). Endoscopic snare resection for tumors of the ampulla of Vater. Yonsei Med J.

[21] Demetriades H, Zacharakis E, Kirou I (2006). Local excision as a treatment for tumors of ampulla of Vater. World J Surg Oncol.

[22] Schlemper RJ, Riddell RH, Kato Y (2000). The Vienna classification of gastrointestinalepithelial neoplasia. Gut.

[23] Norton ID, Gostout CJ, Baron TH, Geller A, Petersen BT, Wiersema MJ (2002). Safety and outcome of endoscopic snare excision of the major duodenal papilla. Gastrointest Endosc.

[24] Bohnacker S, Seitz U, Soehendra N (2005). Endoscopic resection of benign tumors of the duodenal papilla without and with intraductal growth. Gastrointest Endosc.

[25] Singh P, Das A, Isenberg G, Wong RCK, Sivak MV, Agrawal D, Chak A (2004). Does prophylactic pancreatic stent placement reduce the risk of post-ERCP acute pancreatitis? A meta-analysis of controlled trials. Gastrointest Endosc.

[26] Harewood G, Pochron N, Gostout CJ (2005). Prospective, randomized, controlled trial of prophylactic pancreatic stent placement for endoscopic snare excision of the duodenal ampulla. Gastrointest Endosc.

